# Balser Plate Stabilization for Traumatic Sternoclavicular Instabilities or Medial Clavicle Fractures: A Case Series and Literature Review

**DOI:** 10.1111/os.12726

**Published:** 2020-09-06

**Authors:** Wei‐lou Feng, Xiao Cai, Shu‐hao Li, Zi‐jun Li, Kun Zhang, Hao Wang, Jun Zhang, Yang‐jun Zhu, Dong‐xu Feng

**Affiliations:** ^1^ Department of Orthopaedic Trauma Hong Hui Hospital, Xi’an Jiaotong University School of Medicine Xi’an China

**Keywords:** Sternoclavicular joint; Fracture; Internal fixation; Surgery

## Abstract

**Objective:**

This study was performed to observe the effect of internal Balser plate fixation for treating unstable sternoclavicular joints (SCJ) and displaced medial clavicle fractures.

**Methods:**

From April 2009 to September 2016, 17 consecutive patients who underwent open reduction and internal Balser plate fixation for SCJ dislocations or medial clavicle fractures were retrospectively reviewed. There were 11 male and six female patients, with a mean age of 45.6 ± 15.5 years. Standardized treatment procedures consisted of reduction, creating a space posterior dorsal osteal face of the sternal manubrium, an inverted Balser plating, and postoperative immobilization. At follow‐up, plain radiographs were assessed for fracture union, implant loosening, degenerative changes, and joint congruity. Clinical evaluation included: completion of the Disability of the Arm, Shoulder, and Hand (DASH) questionnaire; determination of the Constant and Murley score and visual analog scale (VAS) score; and assessment of intraoperative and postoperative complications.

**Results:**

All patients were followed up, at a mean follow‐up of 20.1 ± 7.9 months, each fracture had a solid union, and each dislocation showed no sign of recurrent dislocation. The mean shoulder forward flexion was 162.9° ± 8.1°. The mean DASH score was 5.2 ± 5.2 points. The mean Constant and Murley joint function score was 93.7 ± 7.9 points, with 15 excellent cases and two good cases. The mean VAS score was 1.1 ± 1.4 points, showing significant improvement compared with the VAS score preoperatively. Postoperative complications included one wound hematoma which was healed after a debridement and one recurrent instability due to hook migration, which underwent revision reconstruction. All patients were satisfied with their treatment outcome at the final follow‐up.

**Conclusion:**

Sternoclavicular joints dislocation or medial clavicle fractures can be treated successfully with Balser plate fixation. This technique permits early functional exercise while preserving the SCJ.

## Introduction

The sternoclavicular joint (SCJ) is a saddle‐type synovial joint comprising the bulbous medial end of the clavicle and the curved notch of the sternum. It is the only real joint connecting the upper limb to the axial skeleton^1^, ^2^. This joint is incongruous and inherently unstable because the clavicular end is bulbous in shape and the clavicular notch of the sternum is curved^3^, ^4^. Shoulder activities can cause SCJ passive movement in three planes, including elevation in the coronal plane during shoulder abduction, flexion, and extension, and 45° of rotation around the longitudinal axis during arm elevation^5^. Most of the SCJ motion occurs between the articular disc and the clavicle^6^. The stability of the SCJ is largely dependent upon the intra‐articular disc and the strong ligaments and muscles surrounding the joint^1^, ^2^. The joint disc serves as a soft cushion and prevents the clavicle displacing locating medially. Ligaments surrounding SCJ include the rhomboid ligament, the interclavicular ligament, and the capsular ligament. The capsular ligament is the strongest ligament resisting superior displacement of the medial clavicle^1^, ^3^, ^5^.

Sternoclavicular joint dislocations are rare, accounting for 1% of all dislocations and 3% of shoulder girdle lesions; in addition, fractures of the medial clavicle account for only 2% to 3% of all clavicle fractures^7^, ^8^. SCJ injuries are often caused by high‐energy trauma, particularly during traffic and sports accidents. A small number of lesions arise from minor trauma, resulting in recurrent dislocations^8^. SCJ dislocations can be classified into anterior and posterior dislocations. Anterior instability is far more common. Posterior SCJ dislocations and medial clavicle fractures are life‐threatening injuries because of their potential to cause mediastinal compression of the retrosternal structures, such as the brachial plexus, pneumothorax, respiratory distress, and vascular injuries^9^, ^10^. SCJ dislocations, especially posterior dislocations, are often missed because of the lack of obvious findings on anteroposterior chest radiographs and the potentially subtle symptomatology. CT is thought to be the most reliable diagnostic method^4^, ^9^. In addition, CT can distinguish true dislocations from proximal fractures.

Most SCJ dislocations and medial clavicle fractures can be treated successfully by closed reduction. However, the displacing forces of the ligaments around the joint often result in recurrent instability, and the prominence of the medial clavicle may cause discomfort. Moreover, long‐term immobilization after conservative treatment can negatively impact patients’ functional exercises and is inconvenient^6^, ^11^. Furthermore, the rate of medial clavicle fracture nonunion after conservative treatment is approximately 8.3%^12^. Thus, operative management is recommended for unstable SCJ and displaced medial clavicle fractures^7^, ^10^, ^11^, ^13^. Many operative procedures have been described for surgical treatments, and reasonably good outcomes have been attained. Such procedures include osteosynthesis with pins, placement of Kirschner wires, wire and plate fixation, medial clavicle resection, and ligament reconstruction^4^, ^7^, ^8^, ^13^, ^14^, ^15^. However, these treatment options might have the risk of damage to retrosternal structures and sacrifice of SCJ micro‐motion, or result in recurrent SCJ instability due to tendon degeneration. There are no biomechanical data available documenting which of these techniques more adequately restores the normal joint kinematics, and operative treatment is still a challenge for surgeons.

These problems with previous operative techniques prompted us to investigate an alternative technique for unstable SCJ dislocations and displaced medial clavicle fractures. The purpose of the current study was: (i) to explore the therapeutic effect of an inverted Balser plate fixation in the treatment of SCJ instability and medial clavicle fractures; (ii) to explore the advantages and disadvantages of this technique; (iii) to compare the outcomes of this study with those of other studies; and (iv) to summarize the exiting limitations and the possible direction for further study.

## Materials and Methods

### *Inclusion Criteria*

Inclusion criteria for this study were: (i) patients with SCJ dislocations or medial clavicle fractures that could not be reduced by conservative treatment and/or appeared prone to recurrence with movement of the shoulder girdle, or persistent pain in which there was a prominent insult to the skin after conservative treatment; (ii) patient treated with an inverted Balser plate fixation; and (iii) a ≥12‐month follow‐up.

### *Exclusion Criteria*

Exclusion criteria for this study were: (i) patients with a poor general condition who could not risk surgery and anesthesia; (ii) patients with lower demand who insisted on conservative treatments; and (iii) patients underwent other types of surgical management (e.g., T‐shaped plating or ligament reconstruction).

### *General Information*

This retrospective study involved 17 consecutive patients who had undergone open reduction and internal fixation for SCJ dislocations or medial clavicle fractures from April 2009 to September 2016 in Hong Hui Hospital, Xi’an Jiaotong University School of Medicine (Xi’an, China). It was approved by the Ethics Committee of Hong Hui Hospital (no. 201803001). All patients agreed to individual clinical details and accompanying images being published and provided written informed consent. The patients’ characteristics are shown in Table 1. All patients had closed injuries. There were 11 male and 6 female patients. Preoperative radiographic templating was performed to identify the exact location of the medial clavicle or fragments. CT and three‐dimensional CT were used to obtain more information on fracture comminution and displacement. Patients with dislocation were classified into anterior and posterior dislocations^16^, while the Edinburgh classification^7^ was used to categorize the medial clavicle fractures.

**TABLE 1:** Patient characteristics

Case	Age (years)	Sex	Side	Mechanism	Type of injuries	Associated injury	Interval (injury to surgery, days)	Follow‐up (months)	VAS	Shoulder flexion	DASH score	Constant and Murley	Complication
1	39	M	R	Traffic accident	Type 1B2 fracture	None	4 days	42	0	160	8.3	87	None
2	55	M	L	Traffic accident	Type 1B2 fracture‐anterior dislocation	None	2 days	16	2	170	3.3	95	Hook migration need a revision
3	35	F	L	Fall	Anterior dislocation	None	3 days	17	0	160	1.7	98	None
4	54	F	R	Fall	Anterior dislocation	Type IV ipsilateral acromioclavicular joint dislocation	4 days	13	4	150	18.3	72	None
5	15	M	L	Sports	Anterior dislocation	None	6 months	23	0	175	0	100	None
6	69	F	R	Fall	Type 1B2 fracture‐posterior dislocation	None	9 days	16	2	150	15	79	None
7	41	M	L	Fall	Anterior dislocation	Lumbar vertebra fracture	18 months	32	3	160	10.9	93	None
8	71	M	L	Traffic accident	Anterior dislocation	Thoracic injury	5 days	30	0	180	0.8	100	None
9	38	F	L	Traffic accident	Anterior dislocation	None	8 days	22	2	170	2.5	100	None
10	45	M	R	Fall	Anterior dislocation	None	5 days	16	0	160	5	94	None
11	51	F	L	Fall	Type 1B2 fracture	None	7 days	18	0	165	0	100	None
12	49	M	R	Traffic accident	Type 1B2 fracture	Thoracic injury, left radial and ulnar fractures	8 days	15	2	160	6.7	91	Wound hematoma
13	40	M	R	Work accident	Type 1B1 fracture	None	2 months	12	0	170	2.5	96	None
14	28	M	L	Fall	Anterior dislocation	None	11 days	19	0	160	1.7	100	None
15	67	F	R	Fall	Type 1B2 fracture	None	4 days	14	1	165	3.3	98	None
16	53	M	R	Traffic accident	Anterior dislocation	None	8 days	21	3	155	5.8	91	None
17	25	M	R	Fall	Anterior dislocation	None	3 days	15	0	160	2.5	98	None
Mean±SD	45.6 ± 15.5							20.1 ± 7.9	1.1 ± 1.4	162.9 ± 8.1	5.2 ± 5.2	93.7 ± 7.9	

Medial clavicle fractures were assessed based on the Edinburgh classification. Acromioclavicular joint dislocation was assessed based on the Rockwood classification.

DASH, disability of the arm, shoulder, and hand; F, female; L, left; M, male; R, right; VAS, visual analog scale.

### *Surgical Technique*

#### *Anesthesia and Position*

After induction of general anesthesia, the patient was placed in the supine position with support between the two scapulae.

#### *Approach and Exposure*

**Figure 1:**
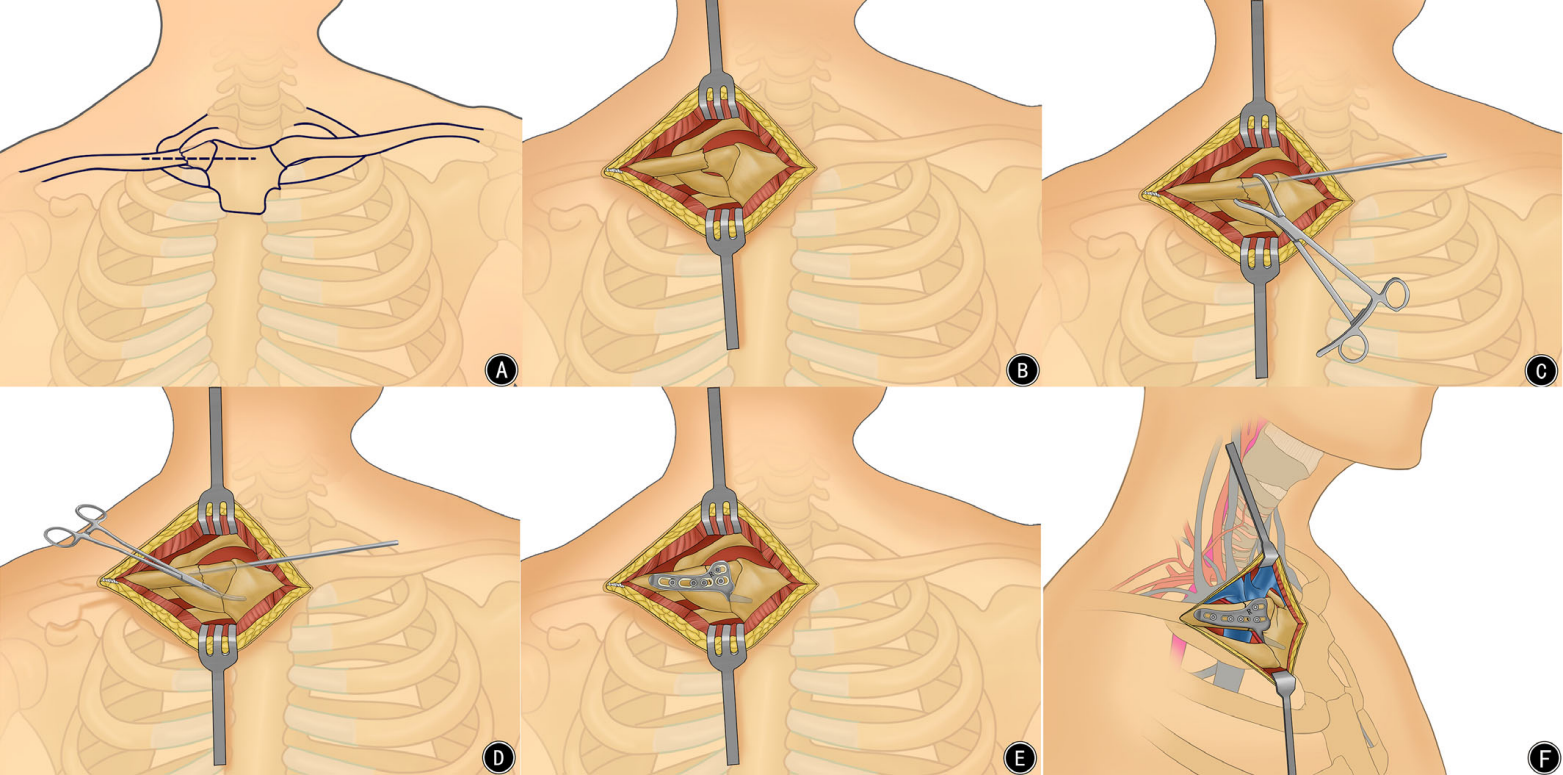
(A) An oblique incision was made starting at the medial one‐third of the clavicle and extending toward the midline of the sternum. (B) Displaced medial clavicle fracture fragments. (C) Using a pointed reduction clamp to achieve fracture reduction. (D) After temporary fixation with a Kirschner wire, a space posterior to the manubrium was bluntly created for the insertion of the Balser plate hook. (E) Accurate reduction and rigid fixation of the medial clavicle and sternoclavicular joint after Balser plating. (F) Relationship between Balser plate and retrosternal structures.

An oblique incision was made starting at the medial one‐third of the clavicle, progressing along the surface of the clavicle and extending toward the midline of the sternum to reveal the sternoclavicular joint, the anterior surface of the sternum, and the medial third of the clavicle (Fig. 1A). The ligaments surrounding the joint could then be divided. These ligaments were always torn in patients with dislocation. After opening the joint capsule, its intra‐articular surface and the clavicle and/or fracture‐displacement were examined (Fig. 1B).

#### *Debridement and Reduction*

Before reduction, the intra‐articular disc was excised in patients with chronic and recurrent dislocations. The incarcerated soft tissue of the sternoclavicular joint was cleaned, and the fracture fragments were debrided. In cases of dislocation, gentle pressure was applied against the medial end of the clavicle with surgeon’s thumb to get the sternoclavicular joint reduction, while in cases of fracture, the reduction was achieved using pointed reduction clamps. Then, 1.5‐mm Kirschner wires were applied for temporary fixation (Fig. 1C). In patients with SCJ dislocation, primary repair of the torn ligaments was performed using nonabsorbable sutures.

#### *Balser Plating*

Before applying the locking Balser plate, a space between the medial head of the clavicle and the first rib posterior dorsal osteal face of the sternal manubrium was bluntly created using curved hemostatic forceps (Fig. 1D). The hook of the plate was shaped to match the contour of the manubrium and then inserted into the space posterior to the manubrium^17^; the other end of the plate was fixed by screws on the anterior part of the medial clavicle (Fig. 1E,F), and Kirschner wires were removed. Finally, the surgical wound was closed in layers.

### *Postoperative Management*

A sling was applied after surgery and used for 4 weeks. Gentle pendulum exercises were commenced at 2 weeks postoperatively, and the frequency of the exercises and the range of motion were gradually increased. Strengthening exercises began at 3 months, and patients were permitted to return to their regular activity without restrictions at 6 months postoperatively.

### *Outcome Measures*

#### *Radiographic Evaluation*

Postoperative follow up including both clinical and radiographic evaluation was performed at 1, 2, and 3 months, and then taken once half‐yearly. In addition, intraoperative and postoperative complications were assessed. Plain radiographs were assessed for fracture union, implant loosening, degenerative changes, and joint congruity. Radiographic fracture union was defined as evidence of at least three of four healed cortices with external bridging of the callus across the fracture, and clinical healing was defined as the absence of functional pain and local tenderness at the fracture site^7^.

#### *Disability of the Arm, Shoulder, and Hand Score*

Upper limb function was evaluated with the completion of the Disability of the Arm, Shoulder, and Hand (DASH) questionnaire, which is scored from 0 to 100. A high score indicates a high level of dysfunction^7^.

#### *Constant and Murley Score*

Shoulder function was evaluated using the Constant and Murley scale,^17^ where a score of between 80 and 100 points was considered an excellent result, a score of between 60 and 79 points was considered good, fair between 40 and 59 points, and poor if <40 points.

#### *Visual Analog Scale*

The pain was evaluated using the visual analog scale (VAS)^14^, which is scored from 0 to 10. A high score represents a high level of pain.

### *Statistical Analysis*

Statistical analysis was performed using SPSS version 17.0 software (SPSS, Chicago, IL, USA). A Wilcoxon matched‐pairs signed rank test was used for VAS analysis, and a *P*‐value of <0.05 was considered statistically significant.

## Results

### *General Data*

The average age at injury was 45.6 ± 15.5 years (range, 15–71 years). The right extremity was injured in nine patients and the left in eight. The mechanism of injury was a fall in nine patients, traffic accident in six, sports injury in one, and work accident in one. Ten patients presented with anterior SCJ dislocations; among them, each case’s medial clavicle was displaced anteriorly by more than one half of the thickness of the clavicle and eight patients presented with a displacement of at least one thickness of its thickness, while two patients had sustained chronic anterior dislocations (more than 3 weeks). Five patients had medial clavicle fractures with the distal segment displaced anteriorly (including one with a chronic fracture) and two patients had fracture‐dislocations (one fracture–anterior dislocation and one fracture–posterior dislocation). Among fracture cases, each distal fracture segment displaced more than half of the thickness of the clavicle. According to the Edinburgh classification^7^, each case was a type 1B fracture, which was defined as a displaced fracture located within the one‐fifth of clavicle length lying medial to a vertical line drawn upward from the center of the first rib; six patients had a type 1B2 (intraarticular) fracture and one had a type 1B1 (extraarticular) fracture.

### *Follow‐up*

At follow‐up, each patient was qualified for the final evaluation. At a mean follow‐up of 20.1 ± 7.9 months (range, 12–42 months), each fracture had solid clinical and radiographic evidence of fracture union, and each dislocation showed no sign of recurrent instability.

### *Physical Function*

At final follow‐up, the mean shoulder forward flexion was 162.9° ± 8.1° (range, 150°–180°). Fifteen patients returned to their pre‐injury daily activities, and all patients were satisfied with their treatment outcome at the final follow‐2).

**Figure 2:**
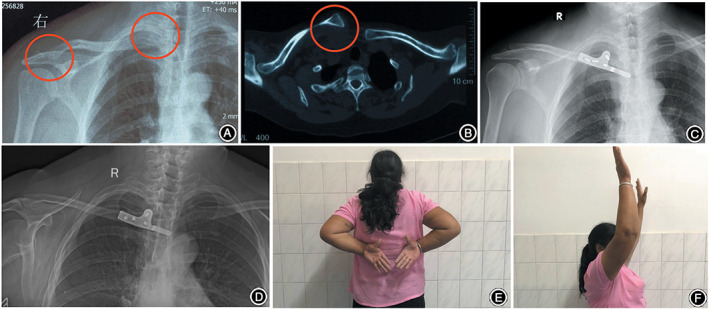
In patient 4 (54‐year‐old woman), the (A) preoperative radiograph and (B) CT scan showed a right anterior dislocation of the sternoclavicular joint (SCJ) and an ipsilateral Rockwood type IV acromioclavicular joint dislocation. (C) A postoperative radiograph after Balser plating showed a good reduction of both the SCJ and acromioclavicular joint. (D) During a follow up of 13 months, the patient showed no recurrent dislocations of the sternoclavicular joint or acromioclavicular joint and developed no hook migration. (E, F) Although the patient had mild shoulder function limitation, she was satisfied with the treatment.

### *Disability of the Arm, Shoulder, and Hand and* *Constant–Murley*

The mean DASH score was 5.2 ± 5.2 points (range, 0.0–18.3 points). The mean Constant and Murley joint function score was 93.7 ± 7.9 points (range, 72–100 points), with 15 excellent cases and two good cases.

### *Visual Analog Scale*

All patients experienced a significant reduction in pain, with a mean VAS score of 1.1 ± 1.4 points (range, 2–7 points) at the final follow up, showing significant improvement compared with the VAS score preoperatively (4.9 ± 1.3) (*P* < 0.05).

### *Complications*

Two postoperative complications occurred in two patients. One patient developed a wound hematoma 3 days postoperatively, which was healed after a debridement. One patient underwent revision reconstruction for recurrent instability due to hook migration 7 days postoperatively. During reconstruction, we applied a screw to fix the medial clavicle and the first rib. The patient showed no sign of further implant migration during follow up and exhibited good function at the final follow up. Other complications like vascular rupture, pleura rupture, and vital organ injury did not occur.

## Discussion

The sternoclavicular joint is a diarthrodial saddle‐type synovial joint and is unstable. Both SCJ dislocations and fractures of the medial clavicle are rare, and there are few established and reliable surgical options^4^, ^10^, ^18^. Although various surgical treatments have been reported for SCJ dislocations and medial clavicle fractures, no gold standard internal fixation equipment suitable for all such injuries has been developed. Considering the proximity between SCJ and important retrosternal structures (trachea, esophagus, brachiocephalic veins, brachiocephalic artery, and brachial plexus), surgical treatment of such injuries is challenging. Kirschner wires are not recommended for the treatment of SCJ dislocations and medial clavicle fractures because of the risk of migration of intact or broken wires into retrosternal structures^14^, ^18^. Some authors prefer ligamentous reconstruction with either sutures or tendons, with the outcomes being satisfactory^13^, ^19^, ^20^, ^21^. However, these strategies all use a bone tunnel in the sternum and the medial clavicle, which may increase the risk of damage to retrosternal structures. In addition, it is hard to treat a very medial clavicle fragment or a comminuted fracture. Other drawbacks may be an early failure because of degeneration, rupture, postoperative immobilization, and elongation, resulting in recurrent SCJ instability^22^. Many surgeons report the successful use of plating (e.g., using a T‐shaped plate or a locking plate) to obtain a rigid fixation between the sternum and the clavicle^7^, ^8^, ^23^. However, as a movement of the shoulder girdle would produce a passive SCJ motion, by allowing early mobilization after surgery, these trans‐articular sternoclavicular techniques might eventually lead to bony erosion around the implants, thereby reducing stability or inducing lytic bone defects^9^. In addition, resection of the end of the clavicle is not frequently recommended because of poor results involving pain and weakness of the upper extremity as well as thoracic outlet syndrome^24^; this procedure should only be considered when all other stabilization options fail or in patients with painful arthroses^25^.

**Figure 3:**
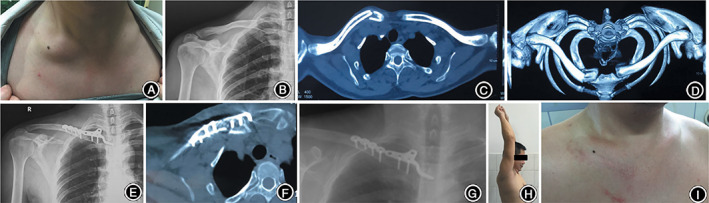
A 40‐year‐old man was injured by machine compression during work 2 months previously. He was treated conservatively at a local hospital and then presented to our institute for further intervention because of persistent pain and a dermal prominence. (A) An obvious dermal prominence was found at the medial end of the clavicle. (B) A preoperative radiograph and (C, D) CT scan showed a displaced right medial clavicle fracture with no obvious bony callus. (E, F) A postoperative radiograph and CT scan showed excellent fracture alignment and fixation with a Balser plate. (G) At 12 months postoperatively, fracture union was present with no implant displacement or breakage. (H) The patient’s full range of motion of the shoulder joint was restored with (I) no pain or dermal prominence.

The technique introduced in the present study was based on previous reports describing the use of a Balser plate for acromioclavicular joint dislocations and distal clavicle fractures, which has been shown to be a very viable method^26^. This is an easy and safe treatment that can be readily mastered by surgeons and has several advantages. First, the technique only requires the creation of a space posterior to the manubrium with simple blunt dissection, which is sufficient for placement of the plate hook in a retrosternal position without risk of damage to retrosternal structures. Second, this dynamic fixation technique permits early mobilization, and it does not damage the cartilage surface of the SCJ. Finally, the two screws near the hook can further improve the stability of the medial end of the clavicle, especially for comminuted medial clavicle fractures (Fig. 3). To our knowledge, only a few papers have described this technique for the treatment of SCJ injuries. In Franck *et al*.^17^ (2003) seven patients were treated with anterior instability and three patients with posterior instability (of which one case was associated with epiphysiolysis) with a Balser plate. Excellent results were achieved without any cases of re‐dislocation. Meanwhile, Qu *et al*. (2019) reported the successful use of this technique to treat 10 patients with anterior sternoclavicular joint dislocation.^5^ However, these previous reports did not focus on the possibility of using this plate to fix medial clavicle fractures, especially periarticular fractures within the medial one‐fifth of the clavicle.

Sabatini *et al*.^20^ published a study evaluating the outcomes of augmented allograft figure‐of‐eight SCJ reconstruction for chronic SCJ instability. In their study, the mean American Shoulder and Elbow Surgeons Shoulder Score increased from 35.3 points preoperatively to 84.7 points postoperatively, the mean VAS score improved from 7.0 before surgery to 1.15 at follow up, and the average Quick‐DASH score was 17.0 points. In contrast, Oe *et al*.^7^ reported the results of 10 medial clavicle fractures treated by different plates. Nine patients attained good functional results with bone union, and one patient underwent resection of the medial two‐thirds of the clavicle because of plate loosening and infection. Eight patients were included in the final assessment, and during a mean follow‐up of 38 months, four patients demonstrated an excellent outcome and three patients demonstrated a good functional outcome. In addition, Liu *et al*.^27^ reported surgical treatment of 11 patients with displaced medial‐end clavicular fractures with reversed lateral locking clavicle plates. The mean DASH score was 8.0 (range, 0 to 13); nine patients were scored as excellent and two patients were scored as good. The outcomes of this study are consistent with the results in the above published studies. At the final follow‐up, 15 patients (88%) returned to full active work at a mean of 7.3 ± 0.7 months postoperatively; the remaining two patients with mild joint function limitation were patients of advanced age with low‐grade physical activity, showing good results with the Balser plate technique for SCJ dislocations and medial clavicle fractures.

This technique also has limitations. First, Balser plating is only indicated for anterior SCJ dislocations or medial clavicle fractures because the hook is inserted posterior to the manubrium, which is contradictory with the direction of the dislocated proximal clavicle. Second, one patient in this study developed hook migration requiring revision. The reason for this might be that the Balser plate can effectively prevent anterior–posterior dislocation but does not easily prevent sagittal plane dislocation. To avoid this, a primary repair of the torn ligaments is advocated before applying the plate during surgery. In addition, the plate should be removed at 6 months postoperatively for patients with dislocations and at 12 months postoperatively for patients with fractures. Third, for chronic and recurrent cases, the intra‐articular disc was excised in this study because it had lost its stabilizing and joint surface‐preserving functions^20^. This has the potential to impact the development of SCJ arthrosis even though degenerative SCJ changes are common and well‐tolerated.

This study had some limitations. It was a small retrospective case series without the use of a control group; biomechanical experiments were lacking. In addition, the follow‐up period was short; we were only able to assess short‐term to intermediate‐term outcomes. However, considering the rarity of SCJ dislocations and medial clavicle fractures and the fact that the study was performed only to discuss the possibility of an alternative fixation technique for unstable SCJ injuries and displaced medial clavicle fractures, the limitations did not influence the results despite the small size of the study. In addition, the findings may be more convincing with a larger sample and comparison with other techniques in the future.

### *Conclusion*

Balser plating was proven to be a safe and effective treatment for SCJ dislocations and displaced proximal clavicle fractures as well as acute and chronic injuries. However, this treatment calls for a well‐trained surgeon to avoid damage to retrosternal structures.
